# The Promoter Structure Differentiation of a MYB Transcription Factor *RLC1* Causes Red Leaf Coloration in Empire Red Leaf Cotton under Light

**DOI:** 10.1371/journal.pone.0077891

**Published:** 2013-10-29

**Authors:** Zhenrui Gao, Chuanliang Liu, Yanzhao Zhang, Ying Li, Keke Yi, Xinhua Zhao, Min-Long Cui

**Affiliations:** 1 Key Laboratory of Pollution Ecology and Environmental Engineering, Institute of Applied Ecology, Chinese Academy of Sciences, Shenyang, China; 2 State Key Laboratory of Cotton Biology, Anyang, China; 3 Institute of Cotton Research of Chinese Academy of Agricultural Sciences, Anyang, China; 4 State Key Laboratory Breeding Base for Zhejiang Sustainable Pest and Disease Control, Institute of Virology and Biotechnology, Zhejiang Academy of Agricultural Sciences, Hangzhou, China; New Mexico State University, United States of America

## Abstract

The red leaf coloration of Empire Red Leaf Cotton (ERLC) (*Gossypium hirsutum* L.), resulted from anthocyanin accumulation in light, is a well known dominant agricultural trait. However, the underpin molecular mechanism remains elusive. To explore this, we compared the molecular biological basis of anthocyanin accumulation in both ERLC and the green leaf cotton variety CCRI 24 (*Gossypium hirsutum* L.). Introduction of R2R3-MYB transcription factor *Rosea1*, the master regulator anthocyanin biosynthesis in *Antirrhinum majus*, into CCRI 24 induced anthocyanin accumulation, indicating structural genes for anthocyanin biosynthesis are not defected and the leaf coloration might be caused by variation of regulatory genes expression. Expression analysis found that a transcription factor *RLC1* (Red Leaf Cotton 1) which encodes the ortholog of *PAP1/Rosea1* was highly expressed in leaves of ERLC but barely expressed in CCRI 24 in light. Ectopic expression of *RLC1* from ERLC and CCRI 24 in hairy roots of *Antirrhinum majus* and CCRI 24 significantly enhanced anthocyanin accumulation. Comparison of *RLC1* promoter sequences between ERLC and CCRI 24 revealed two 228-bp tandem repeats presented in ERLC with only one repeat in CCRI 24. Transient assays in cotton leave tissue evidenced that the tandem repeats in ERLC is responsible for light-induced *RLC1* expression and therefore anthocyanin accumulation. Taken together, our results in this article strongly support an important step toward understanding the role of R2R3-MYB transcription factors in the regulatory menchanisms of anthocyanin accumulation in red leaf cotton under light.

## Introduction

Red leaf cotton is an important genetic resource. The red color produced by anthocyanins is found in most tissues of the plant, except in the cotton fibers; the leaves, stems, petals, and bolls are all red. Accumulation of anthocyanins confers resistance to insect pests such as bollworm, whiteflies, and the boll weevil [1], [2]. Previous genetic studies found that three anthocyanin-related loci – R1, R2, and R_d_ – are responsible for anthocyanin accumulation in red-colored plant species [3]. The R1 locus affects red leaf pigmentation in red leaf cotton including ERLC, the R2 locus is responsible for the red petal spots in green leaf cotton, and the R_d_ locus is involved in dwarf red cotton species [3], [4]. Anthocyanin accumulation in red leaf cotton (ERLC) is sensitized to the light environment such that the red coloration is reduced when plants are grown in shade, whereas plants of the same genotype grown in the field in the summer are solid red in appearance [4]. Red leaf color is a morphological trait that could potentially be useful in cotton breeding. However, the molecular basis of the anthocyanin pigment synthesis mechanism in red leaf cotton remains elusive.

Anthocyanins are flavonoids that are produced in response to a range of environmental and developmental signals. They protect plants against UV light damage, pathogen invasion, and insect attack [5]–[7]. The genetics and biochemistry of the anthocyanin biosynthetic pathway have been well characterized in plants [8]. In *Arabidopsis*, genes participating in the anthocyanin biosynthetic pathway are divided into two groups, early biosynthetic genes (EBGs) including those for chalcone synthase (*CHS*), chalcone isomerase (*CHI*), flavonol 3-hydroxylase (*F3H*), and flavonol 3′-hydroxylase (*F3*′*H*), and late biosynthetic genes (LBGs) including those for dihydroflavonol 4-reductase (*DFR*), anthocyanidin synthase (*ANS*), and anthocyanidin reductase (*ANR*). Each of the two groups has been found to be co-regulated by different regulators [9]–[11]. This gene regulation pattern is similar to that seen in other dicot plants such as *Antirrhinum* and *Petunia*. Anthocyanin biosynthetic pathway genes are predominantly regulated at the transcriptional level, both developmentally and/or in response to various biotic and abiotic stress factors. Many regulatory genes have been identified in different species, for example *AN1* and *AN2* in *Petunia* [12], [13], *C1* and *P1* in *Zea mays* [14], [15], *Rosea1* and *Delila* in *Antirrhinum* [16], [17], *PAP1* and *TTG8* in *Arabidopsis* [18], [19], *MYB1* and *MdMYB10* in apples [20], [21] and *VvMYBA* in grape [22].

The MYB transcription factors constitute the largest transcriptional factor gene family in plants and can be classified into three subfamilies based on the number of highly conserved imperfect repeats in the DNA-binding domain. R3-MYB has one repeat, R2R3-MYB has two repeats, and R1R2R3-MYB has three repeats [23], [24]. Of these, the R2R3-MYB transcription factors have been shown to play important roles in tissue-specific anthocyanin accumulation in many plants; these include *AN2* in *Petunia* [12], *Rosea1*, *Rosea2*, and *Venosa* in *Antirrhinum* [17], *C1* and *P1* in Maize [14], [15], and *PAP1* in *Arabidopsis* [18]. Some mutant varieties display different pigment intensities, also caused by variations in the R2R3-MYB regulators. For instance, a mini-satellite-like structure insertion in the *MYB10* promoter activates its expression resulting in anthocyanin accumulation in the flesh and folium in apples [25]. Mutations in two adjacent MYB regulators, *VvMYBA1* and *VvMYBA2*, inhibit their expression and change the skin color of grape (*Vitis vinifera*) from red to white [26]. Several anthocyanin repressors are also MYB transcription factors, including an R2R3-MYB repressor from strawberry *FaMYB1* [27], *AtMYB4* and *AtMYB6* from *Arabidopsis* [28], *AmMYB308* from *Antirrhinum* [29], and a single repeat MYB gene *AtMYBL2* from *Arabidopsis* [30].

The basic helix-loop-helix (bHLH) transcription factors are another group of regulatory genes involved in anthocyanin biosynthesis in plants. The insertion of a transposon into a bHLH regulatory gene was found to alter the flower color of morning glory [31], and a transposon insertion altered flower color in *Antirrhinum* [16]. bHLH factors also regulate several unrelated biosynthetic pathways, including the development of trichomes and seed coat mucilage [32], [33].

In plants, regulation of the anthocyanin biosynthetic pathway is controlled by a transcriptional activation complex consisting of R2R3-MYB, bHLH transcription factors, and WD40 family proteins [34], [35]. In *Arabidopsis*, four R2R3-MYB regulators (*PAP1*, *PAP2*, *MYB113*, and *MYB114)*, three bHLH regulators (*GL3*, *EGL3*, and *TT8*), and a WD40 regulator (*TTG1*) participate in anthocyanin synthesis regulation by forming a MYB-bHLH-WD40 complex [33], [35]. This complex mainly regulates the expression of LBGs in conjunction with their promoters [36]–[39].

In this study, we evidence functional characterization of an R2R3-MYB transcription factor *RLC1* from Empire Red Leaf Cotton (ERLC). Transcriptional analysis in various tissues indicated that *RLC1* was preferentially expressed in the hypocotyl (stem) and leaves. This expression was influenced by light conditions and correlated with anthocyanin accumulation in the leaves of ERLC. Ectopic expression of *RLC1*in *Antirrhinum* and green leaf cotton CCRI 24 significantly upregulated the expression of genes in the anthocyanin pathway and resulted in anthocyanin accumulation in the hairy roots. Transformation analysis of 35S::*RLC1a* (from the start codon to the stop codon in the DNA sequence of CCRI 24) in *Antirrhinum* revealed anthocyanin accumulation in the transformed hairy roots, suggesting that promoter differentiation might be an effect of the gene transcript in CCRI 24. Transient expression analysis of promoter activity revealed that the *RLC1* promoter in ERLC, which contains two 228-bp fragments forming a direct tandem repeat, normally promoted anthocyanin accumulation when introduced into the leaves of CCRI 24; however, the promoter of CCRI 24, which contains only a single 228-bp fragment in the *RLC1* promoter region did not result in anthocyanin accumulation in the leaves. Therefore, the 228-bp fragment tandem repeat is an important feature of the RLC1 promoter in cotton. Taken together, our results strongly suggest that the *RLC1* transcription factor is induced by light is a major regulatory gene controlling anthocyanin accumulation in cotton leaves.

## Materials and Methods

### Plant Material and Growth Conditions

Seeds of *Antirrhinum majus* JI 7 (provided by Professor Enrico Coen, John Innes Centre, Norwich, UK) and cotton seeds of the ERLC and CCRI 24 varieties (from the Cotton Research Institute, Chinese Academy of Agricultural Science, Anyang, China) were used in this study. The seed coats of the cotton seeds were removed and the seeds of *A. majus* JI7 were surface-sterilized with a brief rinse in 70% (v/v) ethanol followed by a wash in a 2% (v/v) solution of sodium hypochlorite for 10 min, and germinated on solid MS medium in a growth room at 25°C with a 16-h light/8-h dark photoperiod.

For light/shading experiments, 20 10-day old seedlings of both ERLC and CCRI 24 germinated on MS medium were divided into two groups. One group was transferred to a greenhouse in direct light conditions, and other group was transferred to a greenhouse shadowed by a double layer of black shading net. After 30 days in culture, the upper fully opened mature leaves were used to isolate RNA and carry out anthocyanin analysis.

### Quantification of Anthocyanins

The leaves were ground to a fine powder in liquid nitrogen, and 0.3 g of the powder was extracted with 1 mL acidic methanol (1% hydrochloric acid, w/v) at room temperature for 12 h with moderate shaking. After centrifugation at 12,000 rpm for 10 min, 800 µL of the supernatant was added to 4 mL of acidic methanol. The absorbance at 530 and 657 nm was determined using a spectrophotometer (UV757CRT, Shanghai), and the relative level of anthocyanin was calculated using the equation A530 – (0.25×A650) [40]. Each sample was tested three times. Error bars indicate the standard deviation (SD) values of the average anthocyanin content.

### Isolation of Full-length *RLC1* cDNA and Phylogenetic Analysis

Total RNA was isolated from 7-day-old hypocotyl tissues (0.3 g) of ERLC using the SV Total RNA Isolation System combined with RNase-free DNase (Promega, Madison, WI). First-strand cDNA was synthesized from 2 µg of total RNA with the Superscript III First Strand cDNA Synthesis Kit (Invitrogen, Carlsbad, CA) in combination with a PCR library kit (Takara, Shiga, Japan). For the isolation of the anthocyanin-specific MYB transcription factor, a set of degenerate primers (Table S1) were designed from plant MYB cDNA and the conserved DNA-binding domain of R2R3-MYBs was amplified by PCR. Amplified fragments of about 240 bp were subsequently ligated into the pMD19-T cloning vector (Takara) and the insert sequenced using vector M13 primers. Nucleotide sequences were translated into amino acid sequences using DNAMAN (version 6) and compared using the BLAST network service from the National Center for Biotechnology Information (NCBI). To isolate the full-length cDNA of the candidate MYB genes, the 3′-ends were amplified using a combination of the gene-specific primer MYB3SP and the RP primer, and the 5′-end was amplified using the specific primer MYB15SP1 and the CA primer (PCR library kit, Takara,) (Table S1). The 3′- and 5′-RACE PCR products were ligated into the pMD19-T vector and sequenced. Additionally, based on the sequence obtained by RACE-PCR, a set of gene-specific primers, Ghi2-F2 and Ghi2-R2, were designed to amplify the full-length coding sequence using Pfu DNA polymerase (Promega). The amplified fragment was cloned into a pEASY-Blunt vector (named pEASY-RLC1) (TransGen Biotech, Beijing, China) and confirmed as the cDNA sequence. The amplified 744-bp full-length cDNA was named *RLC1* (Red Leaf Cotton 1). This identified coding region of *RLC1* was aligned with published MYB gene sequences involved in the anthocyanin biosynthetic pathway from different plants and a phylogenetic tree constructed using ClustalW (version 2.0) [41] and MEGA version 4.0 [42]. The GenBank accession numbers of these proteins are as follows: AmROSEA1 (ABB83826), AtMYB75 (AAG42001), VvMYBA1 (BAD1897), AtMYB90 (AAG42002), NtAN2 (ACO52470), CsRuby (AFB73909), AmROSEA2 (ABB83827), AmVENOSA1 (ABB83828), AtMYB90 (AAG42002), AtMYB113 (NP_176811), AtWER (NP_196979), AtMYB0 (NP_189430), GhMyb10 (CAD87010), MdMYB10 (ABB84753), MdMYB1 (ABK58136), PhAN2 (AAF66727), PhPHZ (ADW94951), PhDPL (ADW94950), VvMYBA1 (BAD18977), VvMYBA2 (BAD18978), and NtAN2 (ACO52470).

### Construction of pBI35S::*RLC1* and Transformation of *A. majus* and CCRI 24

The full length *RLC1* cDNA coding region was amplified from the pEASY-RLC1 plasmid using an *Sma* I-Ghi2-F2 and *Sac* I-Ghi2-R2 gene-specific primer set (Table S1) in combination with Pfu DNA polymerase (Promega), and then sub-cloned into a pEASY-Blunt vector for confirmation by sequencing. Using the *Sma* I and *Sac* I restriction sites, *RLC1* was substituted for the GUS gene in the pBI121 vector to produce the construct pBI35S::*RLC1*, containing the *RLC1* coding region between the 35S promoter and the CaMv terminator. The pBI35S::*RLC1* binary vector was electroporated into *A*. *rhizogenes* AR1193 cells [43]. AR1193/pBI121 as a negative control, AR1193/pBI35S::*ROSEA1* as a positive control, and AR1193/pBI35S::*RLC1* were used to transformation of 7-day-old CCRI 24 and 4-week-old *A. majus* JI 7 hypocotyls according to the method described by Cui [44].

### Expression Analysis of *RLC1* and Structural Genes of the Anthocyanin Pathway

Total RNA was extracted from 0.1-g samples of roots, 7-day-old hypocotyls, leaves, and mature petals of both ERLC and CCRI 24 varieties, and transformed hair roots of *A. majus* and CCRI 24 using the SV Total RNA Isolation System in combination with RNase-free DNase (Promega). First-strand cDNA was synthesized from 2 µg of total RNA with a Superscript III First Strand cDNA Synthesis Kit (Invitrogen). Semi-quantitative RT-PCR for the genes of involved in this work were carried out using a ABI 2720 machine with 10 ng first cDNA and gene-specific primers (Table S2). The following PCR conditions were used: preliminary denaturation for 5 min at 95°C followed by 30 cycles of denaturation at 94°C for 30 s, annealing at 55–60°C for 30 s, and extension at 72°C for 1 min, with a final extension at 72°C for 10 min. The genes AmUBI of *A. majus* and UBI 7 of cotton were used as positive controls [45]. The RT-PCR experiments were repeated at least three times independently and the PCR products confirmed by sequencing.

### Isolation of *RLC1* Promoter Region

The promoters for ERLC and CCRI 24 were isolated using the Genome Walking kit (Takara) with the gene-specific primers SP1, SP2, and SP3 (Table S1). The PCR-amplified 2.3-kb fragment from ERLC and 2.08-kb fragment from CCRI 24 were ligated to the pMD19-T vector (the resulting constructs were named pMD-Rpro and pMD-Gpro, respectively), and M13 primers were used for sequencing. To isolate the DNA sequence between the start and stop codons, PCR was carried out using two primer sets, GhDNA-F1/GhDNA-R1 for exon 1 and exon 2, and GhDNA-F2/GhDNA-R2 for exon 2 and exon 3, and combined with the LA Tag polymerase (Takara). The products were ligated into the pMD19-T vector and sequenced. The full-length *RLC1* genomic sequence was obtained by compiling partial sequences based on overlapping regions. Analysis of the promoter sequence was performed using the PLACE database [46].

### Transient Analysis of *RLC1* Promoter Region

To compare the promoter activities of R_−pro_ of ERLC and G_−pro_ of CCRI 24, the two promoters were amplified using Pfu DNA polymerase (Promega) and the primers *Hin*d III-ProC-F and *Sma* I-ProC-R. The PCR product was double-digested with the restriction enzymes *Hin*d III/*Sma* I, and the 35S promoter on pBI121 and pBI35S::*RLC1* was replaced with R_−pro_ and G_−pro_ to construct the expression vectors R_−pro_::*RLC1*, G_−pro_::*RLC1*, R_−pro_::*GUS*, and G_−pro_::*GUS*. These vectors were electroporated into *A. tumefaciens* strain GV3101 and used for the transient assay.

To determine the promoter activity of *RLC1*, transient assays were performed using mature leaves of green leaf cotton CCRI 24 by infiltration [47]. After infiltration, the leaves were cultured in a growth chamber at 25°C with a 16-h light/8-h dark photoperiod, illuminated with fluorescent light at an intensity of 100 µmol m^−2^s^−1^. After three days of culture, the leaves were histochemically stained for GUS, or color accumulation was observed by microscopy (Leica S8 APO).

### Histochemical GUS Assay

Infiltrated young leaves with GV3101/R_−_pro::*GUS*, GV3101/G_−_pro::*GUS* and GV3101/pBI121 were stained for GUS according to the method described by Jefferson [48].

## Results

### Phenotypic Characterization of Empire Red Leaf Cotton (ERLC)

**Figure 1:**
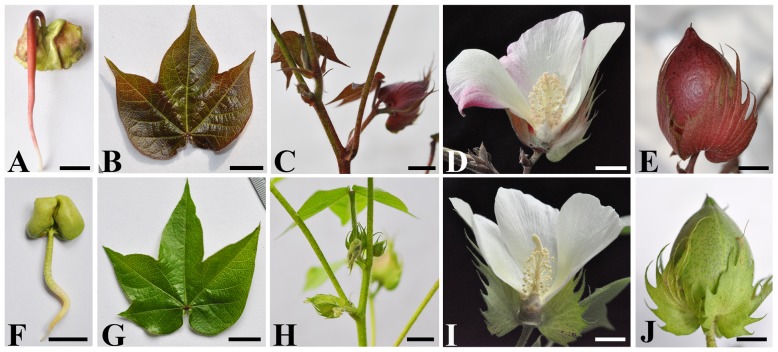
Phenotypic comparison between Empire red leaf cotton (ERLC) (upper panel) and green leaf cotton CCRI 24 (bottom panel) cultivars grown in light. A, A 7-day-old young seedling of ERLC;B, A young leaf from a 4-week-old ERLC plant; C, stems of ERLC; D, A mature flower of ERLC; E, A boll of ERLC; F, A 7-day-old seedling of CCRI 24; G, A young leaf from a 4-week-old plant of CCRI 24; H, stems of CCRI 24; I, A mature flower of CCRI 24; J, A boll of CCRI 24. Scale bar indicates 1 cm.

The ERLC plant exhibits red pigment accumulation throughout its growing period, displaying red coloration in most tissues except the fibers, including seedlings, stems, leaves, calyces, flowers, and bolls when grown in light conditions (Fig. 1A–E). The red color is more intense in the older tissues of the leaves, stems, calyces, and bolls (data not shown); however, the visible color is not apparent in tissues and organs grown in shaded conditions. Additionally, red pigmentation is not observed in fibers in any growth conditions (data not shown). In contrast, the green-leaved cotton cultivar CCRI 24 did not display any color accumulation in seedlings, stems, leaves, calyces, bolls, or fibers, regardless of light conditions (Fig. 1F–J).

**Figure 2:**
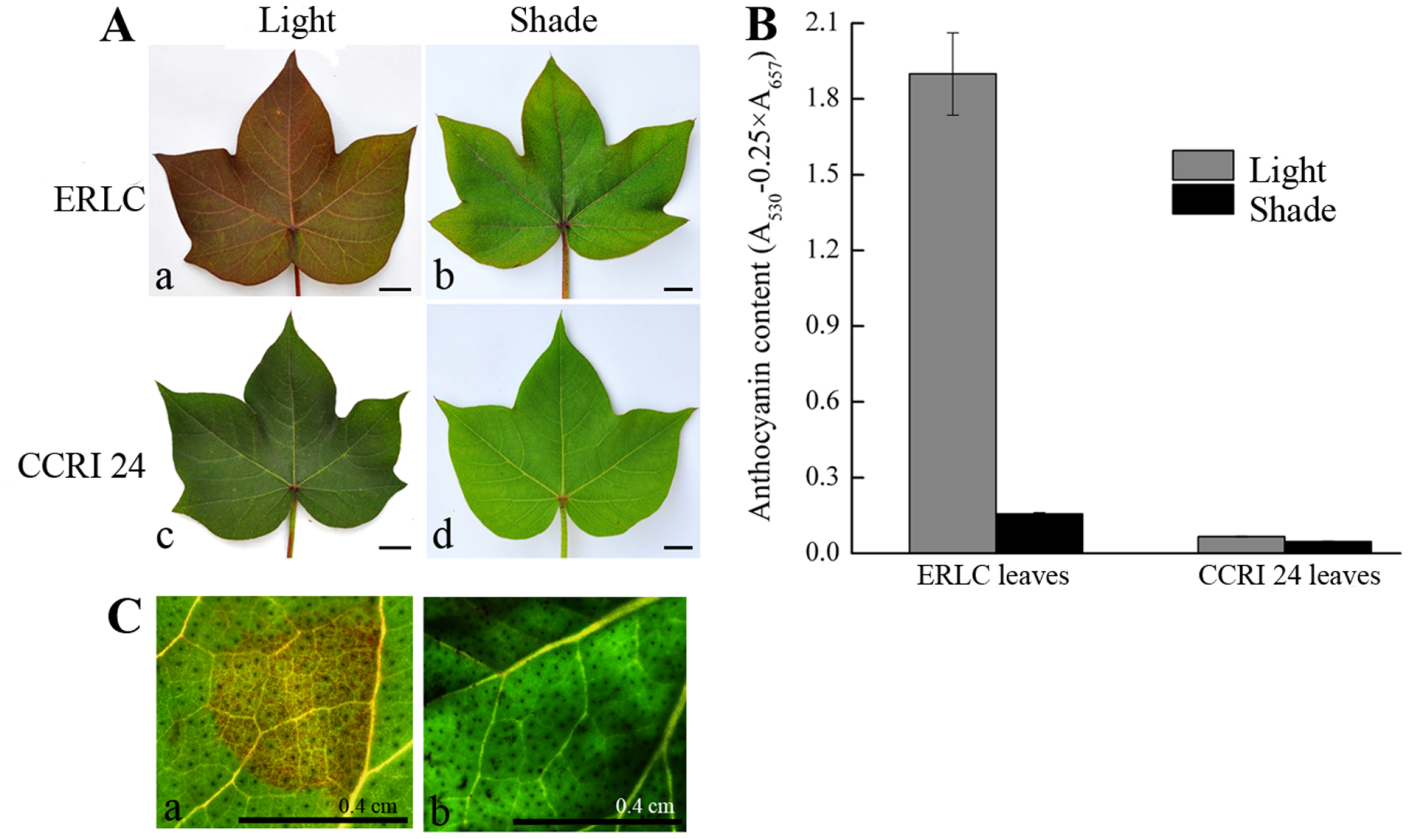
Comparison of leaf colors and analysis of total anthocyanin concentrations of leaves from ERLC and CCRI 24 cultivars grown in light and shade conditions. A, Comparison of leaf colors. The cultivars are indicated on the left and conditions are indicated above. The bar indicates 1; b,A mature leaf of ERLC grown in shade; c, A mature leaf of CCRI 24 grown in light; d, A mature leaf of CCRI 24 grown in shade. B, Total anthocyanin extracted from three fully opened young leaves of each cultivar, respectively, measured using a UV spectrometer. Means of three replicates with error bars indicating standard error (± SD). C, Transient analysis was performed on the leaves of CCRI 24, the *Agrobacterium* strain GV3101/pBI35S::*ROSEA1* (left), and the negative control GV3101/pBI121 (right). The treated cotton leaves were cultured at 25°C in, 16 h light for three days, and observed for color accumulation by microscopy. Scale bar is 0.4 cm.

Previous research found that the red color produced by anthocyanins was distributed throughout the plant in several red leaf cotton cultivars and that the accumulation of this pigment was affected by light conditions [4]. To determine whether the color was indicative of anthocyanin accumulation and whether light conditions affects this accumulation in ERLC, the total anthocyanin content in the leaves of both ERLC and CCRI 24 plants grown in light and shaded conditions was measured using a UV spectrometer (Fig. 2A, 2B). High concentrations of anthocyanins were detected in the leaves of ERLC plants grown in light (Fig. 2A, panel a). However, a lower concentration of anthocyanin was detected in the leaves of ERLC plants grown in shade, and in the leaves of CCRI 24 plants grown in light (Fig. 2A, panels b and c and Fig. 2B). Anthocyanins were barely detectable in the leaves of CCRI 24 plants grown in shaded conditions (Fig. 2A, panel d, and Fig. 2B). Anthocyanin content correlated with light conditions in ERLC plants, with the concentration in light being 11-fold higher than that in shade. However, no significant differences in anthocyanin content were detected between the leaves of CCRI 24 plants grown in light and shade. These results suggest that the light environment strongly affects the anthocyanin accumulation process in ERLC, but that the anthocyanin biosynthesis pathway is not influenced by light conditions in CCRI 24.

Recent studies have detected the expression of structural genes involved in the flavonoid (including anthocyanin) synthetic pathway, such as *CHS*, *CHI*, *F3H*, *DFR*, *ANS*, and *ANR*, in the cotton fibers of several cotton cultivars [49], [50]. Despite no visible anthocyanin accumulation, the expression of structural genes was detected in the leaves of CCRI 24 plants grown in light conditions in our study (data not shown). These results indicate that the expression of structural genes may not directly respond to light and affect anthocyanin accumulation in cotton. Therefore, anthocyanin synthesis in leaves of cotton might be affected by light-induced regulatory genes.

To confirm our prediction, we performed a transient analysis using a combination of mature green young leaves of CCRI 24 and the *Agrobacterium* strain GV3101/pBI35S::*ROSEA1*, harboring the R2R3-MYB transcriptional factor *ROSEA1*, (verified to be a regulatory gene controlling the anthocyanin synthetic pathway in *Antirrhinum)*, under the control of the 35S promoter, by infiltration [47]. Red anthocyanin accumulation was detected in treated leaves of CCRT 24 (Fig. 2C, panel a), but not in GV3101/pBI121, a negative control (Fig. 2C, panel b). This result shows that the expression of *ROSEA1*-like MYB genes affects anthocyanin biosynthesis in the leaves of cotton, and that its expression might be controlled by light.

### Isolation of *RLC1* and Phylogenetic Analysis

Several studies have reported that the R2R3-MYB genes regulate the anthocyanin biosynthetic pathway and that their expression is controlled by light [51], [52]. In this study, we observed color accumulation in the leaves of ERLC in response to light. We designed a degenerate primer set (Table S1) based on the conserved regions in the R2R3 domain of anthocyanin-related MYB transcription factors and performed degenerate PCR to isolate R2R3-MYB transcription factor genes from the cDNA of 7-day-old pigmented hypocotyls of ERLC (Fig. 1A). A 240-bp cDNA fragment was isolated and showed 77% DNA sequence identity with the R2R3 domain of the MYB transcription factor *ROSEA1*, which has been verified to regulate the anthocyanin biosynthetic pathway in *Antirrhinum* [17]. Full-length cDNAs were determined using 5′/3′-RACE PCR techniques. The isolated 744-bp candidate cDNA encodes a protein of 247 amino acids. We named this gene *RLC1* (Red Leaf Cotton 1).

**Figure 3:**
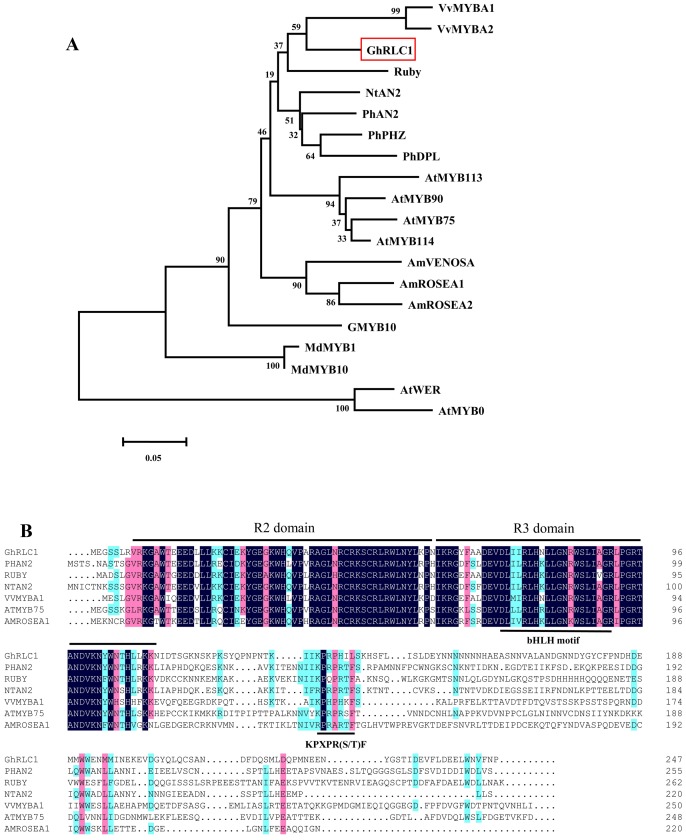
Comparison of the deduced amino acid sequence of RLC1 with verified MYB genes of other plant species. A, Phylogenetic tree of *RLC1* and selected R2R3-MYBs from other plant species. The multiple sequence alignment was performed with the R2R3 domain of MYB proteins. The tree was constructed using the neighbor-Joining method using MEGA software. Numbers along the branches indicate bootstrap support determined from 1,000 trials, and the bar indicates an evolutionary distance of 0.05%. B, Alignment of deduced amino acid sequences of RLC1 with MYB transcriptional regulators. The R2 and R3 repeat domains are indicated by lines above, and the conserved region of the bHLH interacting motif ([DE]Lx2[RK]x3Lx6Lx3R) and the conserved KPRPR[S/T]F motif are underlined.

Phylogenetic analysis showed that RLC1 belonged to the same cluster as VvMYB1, VvMYB2, and Ruby, which have been shown to regulate the anthocyanin synthesis pathway in grape species and in *Citrus sinensis* (Fig. 3A). The deduced amino acid sequence of RLC1 shared 85% and 86% identity with VvMYB1 and Ruby, respectively, in the R2R3-MYB DNA-binding domain. The completed protein sequence of RLC1 was 44% identical to VvMYB1 of *grape*, and 46% identical to Ruby of *Citrus sinensis*. The alignment of the RLC1 protein sequence with some known R2R3-MYB regulators from different plant species revealed a very conserved R2R3 repeat domain and a [DE]Lx2[RK]x3Lx6Lx3R motif for interaction with bHLH proteins (Fig. 3B). Additionally, another signature motif, KPXPR(S/T)F, which is specific to MYB proteins that activate anthocyanin biosynthesis, was found [53]. Thus, sequence analysis indicated that *RLC1* is an R2R3-MYB like gene involved in the regulation of the anthocyanin synthetic pathway in ERLC.

### Functional Analysis of *RLC1*

**Figure 4:**
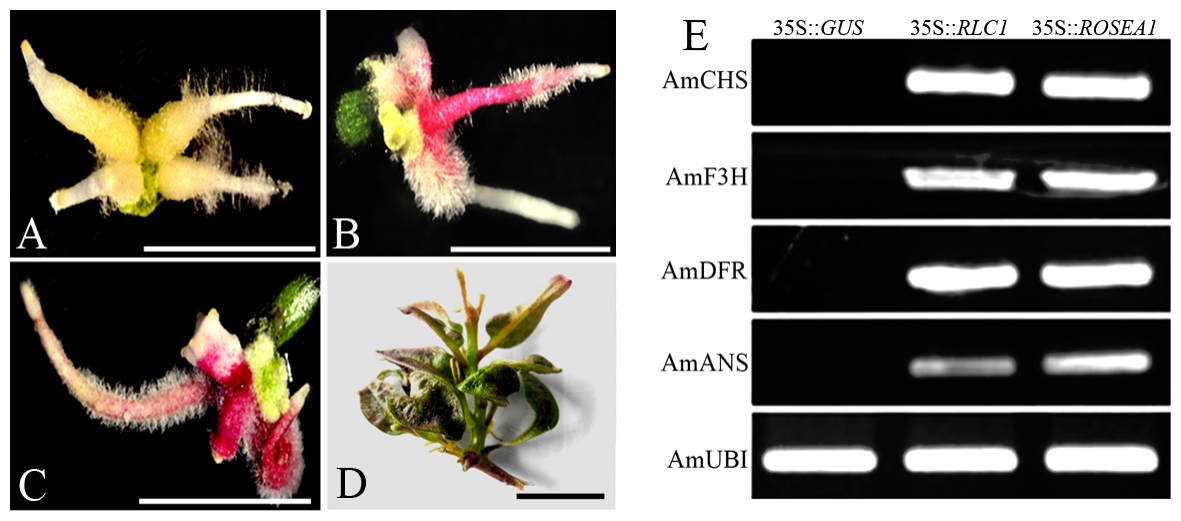
Phenotypic comparison of the hairy roots of *A. majus* transformed with pBI121, pBI35S::*ROSEA1*, and pBI35S::*RLC1*, and expression analysis of genes involved in the anthocyanin pathway in *A. majus by* RT-PCR. Hairy roots transformed with A, pBI121; B, pBI35S::*RLC1*; C, pBI35S::*ROSEA1*. D, A shoot regenerated from hairy roots transformed with pBI35S::*RLC1.* Anthocyanin pigmentation is apparent in the leaves and stems. E, RT-PCR analysis of gene expression in the hairy roots of *A. majus* transformed with pBI121 (negative control), pBI35S::*ROSEA 1* (positive control), and pBI35S::*RLC1*, respectively.

To determine the function of *RLC1*, an expression binary vector pBI35S::*RLC1*, harboring the full-length *RLC1* cDNA under the control of the cauliflower mosaic virus 35S promoter, was transformed into hypocotyl segments of *A. majus* JI7 by *A. rhizogenes*-mediated transformation [44]. pBI35S::*ROSEA1* as a positive control and pBI121(35S::*GUS*) as a negative control were also transformed into *A. majus*. Two weeks after infection, transformed non-pigmented hairy roots emerged from the wounded end of hypocotyl segments of *A. majus* co-cultivated with pBI121, pBI35S::*CRL1*, and pBI35S::*ROSEA1* (Fig. 4A). Highly pigmented hairy roots emerged from hypocotyl segments transformed with pBI35S::*RLC1* and pBI35S::*ROSEA1* later than non-pigmented hairy roots. Pigmented hair roots were also found to elongate slower than non-pigmented hairy roots (Fig. 4B, 4C). The callus, hairy roots, and emerged shoots of segments transformed with pBI121 showed no pigmentation. Additionally, we obtained 11 and 14 independent pigmented hairy roots of pBI35S::*RLC1* and pBI35S::*ROSEA1*, respectively, after four weeks of culture. Transformed shoots from pigmented hairy roots consistently showed pale or dark red pigmentation in the leaves and stem (Fig. 4D).

To confirm that *RLC1* activates genes of the anthocyanin biosynthetic pathway, we investigated the expression levels of the structural genes *CHS*, *F3H*, *DFR*, and *ANS* in the hairy roots of *A. majus* of transformed with pBI121, pBI35S::*RLC1* or pBI35S::*ROSEA1* by semi-quantitative reverse transcription (RT)-PCR. No visible expression of the investigated structural genes was detected in negative control hairy roots transformed with pBI121. However, the expression of structural genes was dramatically up-regulated in hair roots transformed with both pBI35S::*RLC1* and pBI35S::*ROSEA1* (Fig. 4E). *RLC1* and *ROSEA1* activated the expression levels of these structural genes to very similar extents, and this correlated with anthocyanin accumulation in hairy roots transformed with pBI35S::*RLC1* and pBI35S::*ROSEA1* (Fig. 4). These results indicate that *RLC1* has similar functions to *ROSEA1* of *A. majus* and that it is commonly involved in regulating the anthocyanin synthetic pathway in ERLC.

### Analysis of *RLC1* Expression Patterns between ERLC and CCRI 24

**Figure 5:**
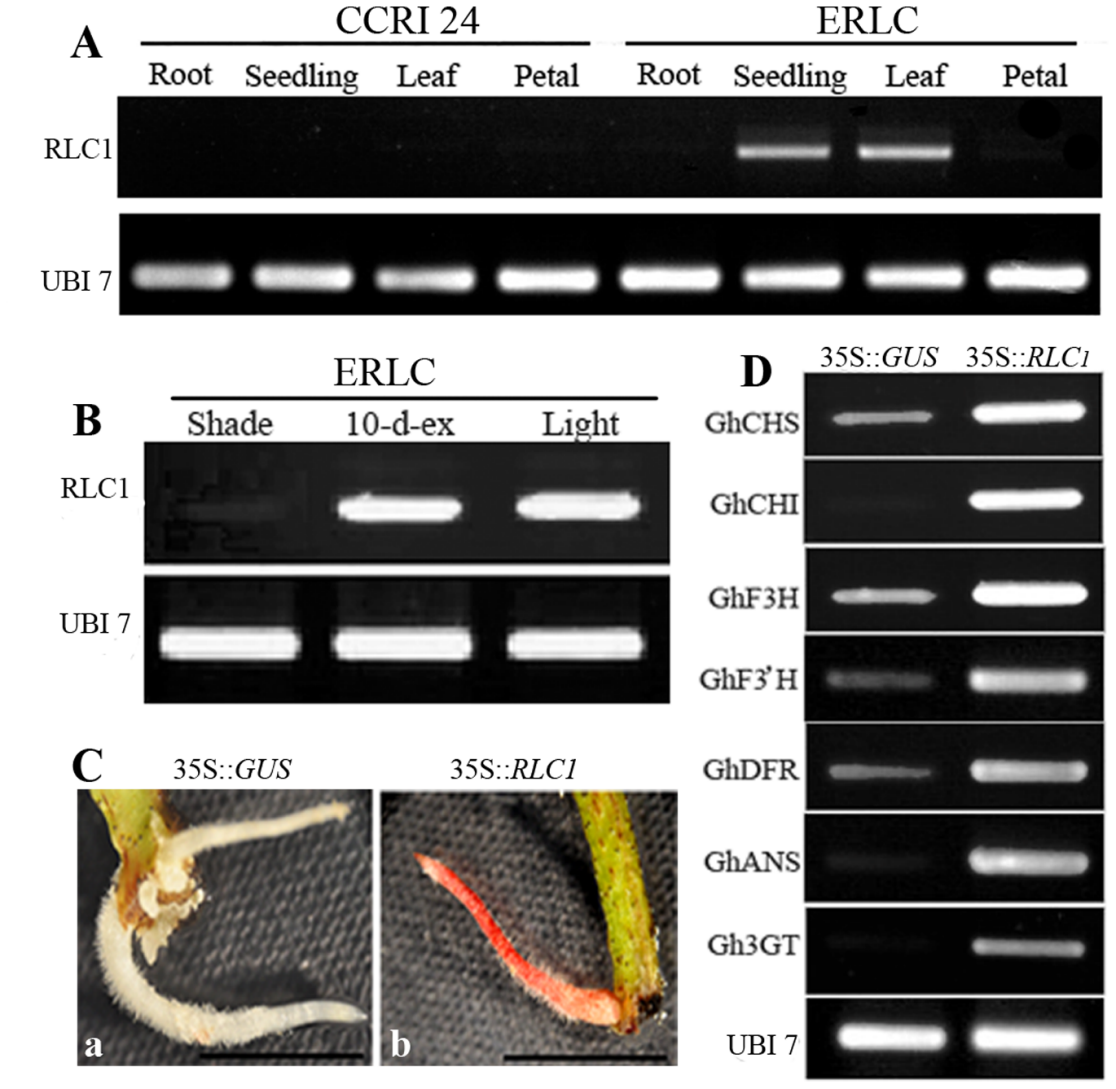
Analysis of *RLC1* and gene expression levels in cotton by RT-PCR. UBI 7 was used as a positive control. **A**, *RLC1* expression analysis was performed in roots, seedlings, leaves, and mature petals of ERLC and CCRI 24 cultivars grown in light by semi-quantitative RT-PCR. **B**, Comparison of the expression levels of *RLC1* in mature leaves of ERLC grown in shade, light, and combined conditions. **C**, Comparison of color accumulation in transformed hairy roots of CCRI 24. **a**, Hairy root transformed with pBI121; **b**, Hairy root transformed with pBI35S::*RLC1.* Scale bar indicates 1 cm. **D**, Expression analysis of structural genes in hairy roots of transformed CCRI 24 with the negative controls pBI121 and pBI35S::*RLC1*, respectively.

To investigate and compare *RLC1* expression between the ERLC and CCRI 24 varieties, semi-quantitative RT-PCR analysis was performed using total RNA isolated from roots, seedlings, leaves, and mature petals. Both ERLC and CCRI 24 plants were grown in light conditions. The expression pattern of *RLC1* was compared in ERLC and CCRI 24 (Fig. 5A). Strong expression of *RLC1* was detected in the seedlings and leaves of ERLC, but *RLC1* was barely expressed in the roots and petals of ERLC. Slight expression of *RLC1* was detected in the leaves of CCRI 24, but expression of *RLC1* was silenced in the roots and seedlings of CCRI 24.

To investigate the effect of light conditions on the expression level of *RLC1*, RT-PCR analysis was performed using total RNA isolated from the leaves of ERLC plants grown in light, those grown in the shade, and those transferred to light after 10 days in the shade (Fig. 5B). Slight expression of *RLC1* was detected in the leaves of plants grown in the shade. Remarkably up-regulated expression of *RLC1* was detected in the leaves of plants 10 days after being transferred from shade to light. The expression level was very similar to that in leaves grown in light (Fig. 5B). The *RLC1* transcript level coincided with anthocyanin content and coloration in ERLC and CCRI 24 cultivars (Fig. 2A, 2B; Fig. 5B). These results indicate that light-induced *RLC1* regulates anthocyanin accumulation in the leaves of ERLC plants. Furthermore, reduced expression of *RLC1* may cause the absence of pigmentation in the leaves of CCRI 24 plants.

To test our hypothesis, pBI35S::*RLC1* and the negative control plasmid pBI121 were introduced into 7-day-old hypocotyls of CCRI 24 by *A. rhizogenes*–mediated transformation. After six weeks of infection, five pigmented hairy roots were obtained from 30 hypocotyl segments transformed with pBI35S::*RLC1.* Hairy roots from plants transformed with pBI121 did not show any colors (Fig. 5C). To investigate the role of *RLC1* in the anthocyanin biosynthetic pathway in CCRI 24, we analyzed the expression levels of the structural genes *CHS*, *CHI*, *F3H*, *F3′H*, *DFR*, *ANS*, and *3GT* in hairy roots transformed with pBI12 and pBI35S::*RLC1* by semi-quantitative RT-PCR. All the investigated structural genes were expressed in negative control hairy roots transformed with pBI121; in particular, *CHI* and *3GT* were only slightly expressed. In pigmented hairy roots transformed with pBI35S::*RLC1*, *RLC1* clearly activated the expression of the investigated structural genes, with *CHI* and *3GT* expression being significantly up-regulated (Fig. 5D). These findings clearly indicate that all the structural genes involved in the anthocyanin biosynthetic pathway in CCRI 24 were normal. The expression level of *RLC1* influences the transcription of structural genes and affects anthocyanin accumulation in the leaves of CCRI 24 plants. Differential expression patterns of *RLC1* between ERLC and CCRI 24 might be due to differences between alleles.

### Comparison of *RLC1* Alleles between ERLC and CCRI 24

**Figure 6:**
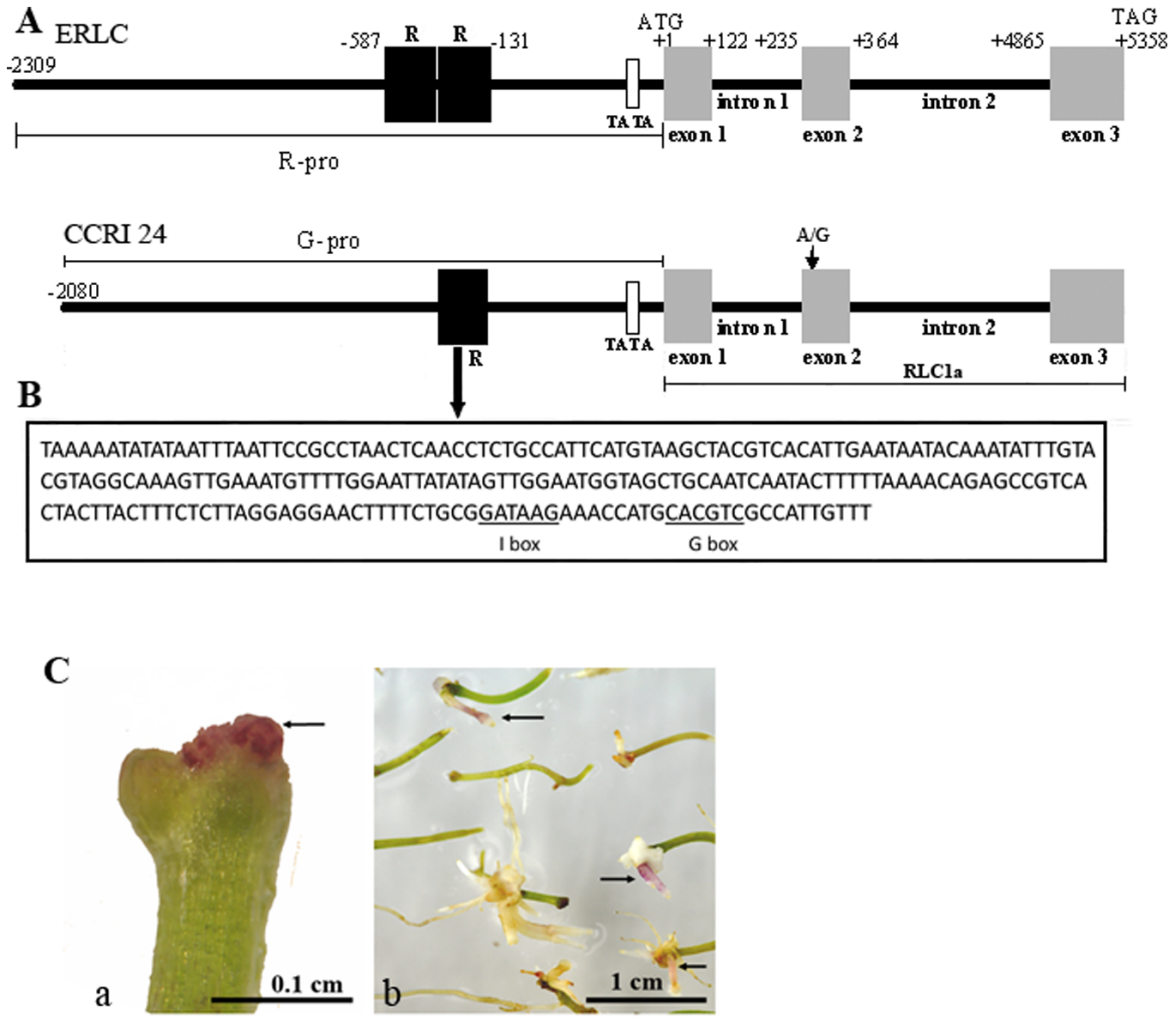
Comparison of the *RLC1* alleles between ERLC and CCRI 24. A, The coding sequence is shown in gray boxes and the non-coding sequence is shown as a black line. Location of the putative TATA box is shown by an empty square. Repeat fragments (R) are indicated by black squares. The exons, introns, and promoter region are labeled. The location of a nucleotide change site is also indicated in exon 2 of CCRI 24. Numbers refer to the position relative to the first nucleotide of the start codon. B, DNA sequence of the 228-bp fragment, with the location of putative I-box and G-box (underlined). C, Functional analysis of the RLC1a region of CCRI 24 (Fig. 6A, bottom) in the hairy roots of *A. majus* by *A. rhizhogenes-*mediated transformation. The pBI35S::RLC1a expression vector contained the RLC1a region on pBI121 driven by the cauliflower mosaic virus 35S promoter. a, a red pigmented mass developed on the end of hypocotyl segment of *A. majus* two weeks after infection; b, red pigmented hairy root developed from the ends of hypocotyl segments four weeks after infection. The pigmented mass (a) and hairy roots (b) are indicated by small arrows.

To test this idea, a 7-kb DNA fragment was isolated from ERLC genomic DNA using a DNA Walking Kit. This fragment included a 2.3-kb promoter region, three exons, and two intron regions (Fig. 6A, top). Based on the 7-kb DNA sequence of ERLC, a corresponding DNA fragment was amplified from the CCRI 24 genomic DNA by PCR (Fig. 6A, bottom). On comparing the DNA coding regions of ERLC and CCRI 24, we found that the CCRI 24 sequence also showed the same structural features as that of ERLC, with the same lengths for exon 1, exon 2, exon 3, intron 1, and intron 2. We found thirteen nucleotide polymorphisms in the introns (data not shown) and 1 nucleotide difference in exon 2 where an adenine (A) instead of a guanine (G) in exon 2 resulted in an Arg-to-His change at the R2 repeat domain of the deduced amino acid sequence (Fig. 6A, bottom). Except for an amino acid change, the coding region can be encoded as a full-length *RLC1* cDNA with 99.6% identity between ERLC and CCRI 24. A major difference between ERLC and CCRI 24 was determined in promoter regions. The 2.3-kb promoter region of ERLC (R_−pro_) has a putative TATA box at position −89 and two 228-bp length repeat fragments (R) located in sequence between positions −131 and −587. However, the promoter region of CCRI (G_−pro_) is shorter than R_−pro_ by 228 bp and lacks one of the repeats (R) at position −359 to −588. We also analyzed the 228-bp fragment sequence in the Plant Cis-acting Regulatory DNA Elements (PLACE) database [46], and found a putative I-box and a G-box that have been identified by several groups as a cis-acting element involved in the activation of light-regulated plant genes [54], [55]. However, we did not find other putative cis-acting elements or structural candidates in the sequence of the 228 bp-fragment.

### The Two 228-bp Fragments Form an Important Motif in the *RLC1* Promoter

To detect the effects of the thirteen polymorphisms in the introns and the amino acid change for *RCL1* transcription in CCRI 24, we created an expression vector pBI35S::*RLC1a*, harboring a 5-kb DNA fragment including the three exons and two introns of CCRI 24(Fig. 6A, bottom), under the control of the cauliflower mosaic virus 35S promoter. This expression vector was transformed into hypocotyls of *A. majus* by *A. rhizogenes*-mediated transformation. Two weeks after infection, we observed a red colored mass growing from the end of the hypocotyl, and several red-pigmented hairy roots were obtained four weeks after culture (Fig. 6C). This result indicated that the amino acid change and thirteen polymorphisms in the introns did not affect *RLC1* transcription in CCRI 24. This suggests that the differences in the promoter region between ERLC and CCRI 24 might affect *RLC1* expression (Fig. 6A).

**Figure 7:**
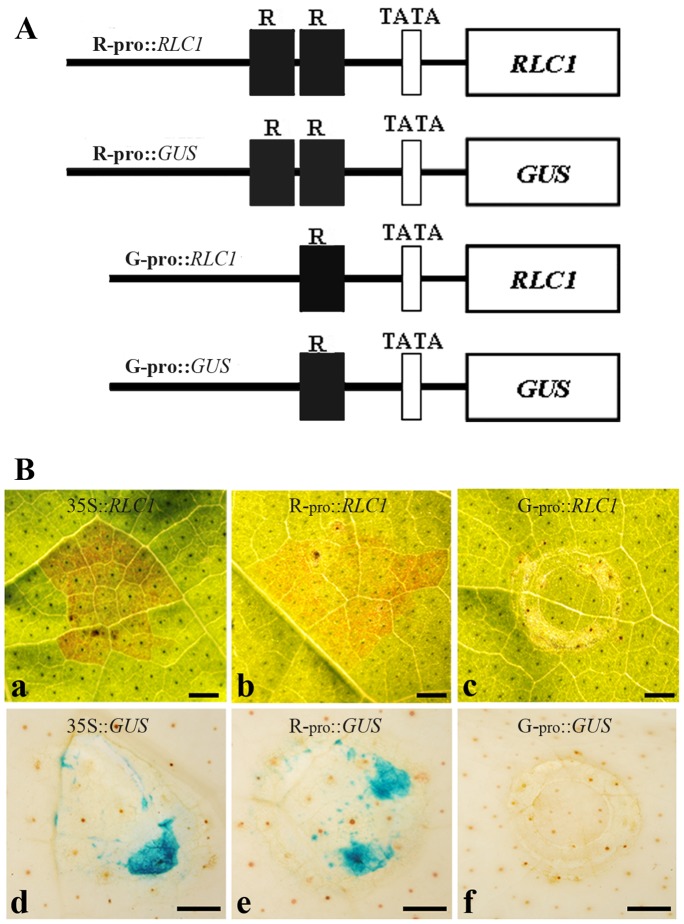
Analysis of *RLC1* promoter activity by infiltration. **A**, Diagrams of constructs for the analysis of *RLC1* promoter activity. R_−pro_: 2300-bp promoter region of *RLC1* of ERLC; G_−pro_: 2080-bp promoter region of *RLC1* of CCRI 24 (Fig. 6). **B**, promoter activity tests were performed using mature young leaves of CCRI 24 using the combination of expression vectors described above. Treated cotton leaves were cultured at 25°C with 16-h light periods for three days, and the leaves were used for color observation or GUS staining. **a**, A leaf infiltrated with 35::*RLC1* and cultured for three days in light; **b**, A leaf infiltrated with R−pro::*RLC1* and cultured for three days in light; **c**, A leaf infiltrated with G_−_pro::*RLC1* and cultured for three days in light; **d**, A GUS-stained leaf infiltrated with pBI121 and cultured for three days in light; **e**, A GUS stained leaf infiltrated with R−pro::*GUS* and cultured for three days in light; **f**, A leaf infiltrated with G_−_pro::*GUS* and cultured for three days in light. Scale bar is 0.1 cm.

To confirm our expectation, we created several expression vectors R−_pro_::*RLC1*, R−_pro_::*GUS*, G_−pro_::*RLC1*, and G_−pro_::*GUS* (Fig. 7A) and performed transient analysis using young mature green leaves of CCRI 24 with an infiltration method [47]. At three days post-infiltration, red color was observed at the infiltrated points in cotton leaves with R−_pro_::*RLC1* and 35S::*RLC1* respectively (Fig. 7B, panels a and b). However, the red color was not observed in leaves infiltrated with G_−pro_::*RLC1* (Fig. 7B, panel c). Similar results were also observed in the transient analysis using the β-glucuronidase (GUS) gene as the reporter, driven by the R−_pro_ or G_−pro_ promoter (Fig. 7B, panels d–f). These experiments strongly support the hypothesis that the repeat structure is necessary as a putative donor motif on the promoter to regulate *RLC1* expression in cotton in light conditions.

## Discussion

### *RLC1* is an R2R3-MYB Transcription Factor

In this study, using degenerate PCR based on a R2R3-MYB domain-specific sequence, we isolated a transcriptional factor, named *RLC1*, from ERLC. *RLC1* encoded a R2R3-MYB protein clearly placed in a clade with many anthocyanin-regulating MYB transcription factors from different species such as *PhAN2* [12], Rosea1 [17], PAP1 [18], MdMYB1 [51], Ruby [56], VvMYBAs [22], [26], [57] and NtAN2 [58]. Alignment of the amino acid sequence of RLC1 showed significant similarity to structures with Ruby and PhAN2, which harbored very conserved R2R3 domains, a conserved ([DE]Lx2[RK]x3-Lx6Lx3R) motif thought to be responsible for the interaction between bHLH and MYB proteins (Fig. 3) [59], and a KPRPR[S/T]F motif specific to R2R3-MYB regulators in anthocyanin synthesis [53]. Genomic DNA sequence analysis also showed that *RLC1* has three exons. The R2 domain spans exons 1 and 2, and the R3 domain spans exons 2 and 3. The genomic organization and R2R3 domain distribution of *RLC1* was similar to that of other R2R3-MYBs [12], [18], [58]. These structural similarities in the DNA and amino acid sequences suggest that *RLC1* is likely to have similar functions to the anthocyanin -specific MYB regulators and a common evolutionary origin.

### *RLC1* is *PAP1/ROSEA1/*Ortholog Gene

In this study, we confirmed that *RLC1* is a positive regulator of anthocyanin synthesis through the transformation of *Antirrhinum* and green leaf cotton CCRI 24. Overexpression of *RLC1* resulted in anthocyanin accumulation in the transformed hairy roots of *Antirrhinum* and cotton.

In the hairy roots of *Antirrhinum*, 35S::*GUS* did not affect expression of the endogenous anthocyanin synthesis genes *Am CHS*, *AmF3H*, *AmDFR*, and *AmANS*, and the hairy roots lacked coloration. However, the expression of *Am CHS*, *AmF3H*, *AmDFR*, and *AmANS* was significantly upregulated by 35S::*RLC1*, and strong anthocyanin accumulation was observed in the hairy roots in contrast to those of plants transformed with 35S::*GUS*, which lacked anthocyanin. 35S::*RLC1*-activated expression levels of anthocyanin synthetic genes showed patterns similar to those activated by 35S::*ROSEA1* in transformed hairy roots (Fig. 4). These results suggest that *RLC1* is an ortholog of *ROSEA1* in *Antirrhinum*, and is mainly involved in the regulation of anthocyanin synthesis in cotton.

In cotton, despite the negative control in hairy roots transformed with 35S::*GUS* that lacked anthocyanin accumulation, the endogenous structural genes *GhCHS*, *GhCHI*, *GhF3H*, *GhF3′H*, *GHDFR*, *GhANS*, and *Gh3G*T in the anthocyanin pathway showed detectable expression. Recent studies have detected the expression of these structural genes in white fibers [49], [50]. Therefore, we determined which structural genes are commonly expressed in most tissues and organs of green leaf cotton, and whether they might be involved in the production of other flavonoids, such as proanthocyanidins. We also found that the structural genes *GhCHI* and *Gh3GT* were significantly upregulated in the transformed hairy roots of 35S:*:RLC1* as well; these hairy roots showed notable anthocyanin accumulation (Fig. 5C, 5D). Therefore, the expression levels of *GhCHI*, an EBG, and *Gh3GT*, an LBG, may affect anthocyanin biosynthesis and which genes expression is controlled by *RLC1* expression. Although the R2R3-MYB genes mostly regulate LBG expression in the flavonoid pathway [17], similar disagreements regarding the regulation of EBGs and LBGs have been reported in the over-expression of *PAP1* in transgenic *Arabidopsis* and *NtAN2* in tobacco. Over-expression of the *PAP1* and *NtAN2* MYB regulators results in the up-regulation of genes across the entire phenylpropanoid pathway in *Arabidopsis* and tobacco [35], [58]. Therefore, *RLC1* and *PAP1* may redundantly regulate the transcription of EBGs in a manner similar to MYB regulators in *Arabidopsis* and cotton. Additionally, the elevated expression of EBGs may be related to a metabolite feedback phenomenon, induced by the up-regulation of LBGs.

Since the transcription of structural genes in the anthocyanin pathway was affected, the genetic basis of cotton color is probably due to the activity of a common regulator of the *RLC1* gene. This would be analogous to *PAP1* in *Arabidopsis* and *ROSEA1* in *Antirrhinum* [17], [18].

### Promoter Differentiation Affects *RLC1* Expression between ERLC and CCRI 24

In this study, we investigated *RLC1* alleles between red leaf cotton ERLC and green leaf cotton CCRI 24. We found a 228-bp fragment spanning positions −131 to −359 bp in the *RLC1* promoter of CCRI 24 (G_−pro_), and two 228-bp fragments were detected from position −131 to −587 bp forming a near-perfect direct tandem repeat in the *RLC1* promoter of ERLC (R_−pro_). Further, through transient expression analysis of *RLC1* promoter activity in both ERLC and CCRI 24, we found out that the promoter of ERLC (R−_pro_) normally drove fused genes (Fig. 7). These results suggested that the repeat structure is a putative donor motif of the promoter region that acts to promote *RLC1* gene expression in leaves of cotton.

### Light Stimulation Causes *RLC1* Expression in ERLC

Environmental conditions control the anthocyanin biosynthetic pathway in plants. In *Arabidopsis*, maize, apple, and grape, anthocyanin biosynthesis is enhanced by sunlight stimulation [39], [53], [60]–[63]. In mature grape plants, light and sugar content affect MYB regulation of flavonoid biosynthesis [61]. Furthermore, in apple, MYB activation of anthocyanin synthesis is affected by light. When the fruit are bagged, MdMYB1 expression is prevented in the red-skinned apple “Cripps Pink” [51]. High light exposure also upregulates the *Arabidopsis* R2R3-MYB activators *PAP1* and *PAP2* and their bHLH partner *TT8* [39], [64]. Moreover, it has been previously reported that the expression of specific maize *MYB* genes is strongly light induced and is closely correlated with the light induction of structural genes and anthocyanin biosynthesis [60], [64].

In this study, we have shown that anthocyanin biosynthesis in the leaves of cotton plant is also controlled by light. The expression level of *RLC1* and anthocyanin accumulation in leaves of ERLC was affected by light conditions (Fig. 2; Fig. 5B). When grown in light, *RLC1* was strongly expressed in leaves and seedlings. However, when grown in shaded conditions, *RLC1* was scarcely expressed in leaves, but when plants were transferred to light, expression of *RLC1* recovered to normal levels and the leaves developed a deep red color (Fig. 5B). The *RLC1* expression pattern also correlated with the expression of other anthocyanin biosynthetic genes.

In addition, we also analyzed the 228-bp fragment sequence against the PLACE database [46], and found several putative cis elements. Among them, we detected a putative I-box and G-box identified by several groups as a cis-acting element involved in the activation of light-regulated plant genes [54], [55], [65]. Therefore, a light signal might interact with a light-responsive element in the promoter region of the *RLC1* gene, and enhance the expression of *RLC1*, which would then regulate the expression of anthocyanin structural genes and the anthocyanin pathway in cotton. Although most genes in the anthocyanin biosynthesis pathway are known, it is not known how the MYB genes regulate anthocyanin biosynthesis in cotton. We have shown that the R2R3-MYB gene *RLC1* regulates anthocyanin structural genes in response to light induction. However, the influence of other bHLH and WD40 cofactors required for anthocyanin synthesis have not been determined and so their role in light regulation remains to be determined.

In summary,in ERLC the anthocyanin pathway is constitutively regulated but is also responsive to light. We have identified an R2R3-MYB transcriptional factor *RLC1* that has an expression pattern correlating with that of anthocyanin accumulation in the leaf and in response to light. The MYB regulator *RLC1* can enhance the expression of structural genes in transformed hairy roots of *Antirrhinum* and cotton. This expression is due to a motif of two 228-bp fragments forming tandem repeats more than −131 bp upstream of the promoter region of *RLC1*. These results suggest that *RLC1* regulates by light induction structural genes across the anthocyanin pathway in red leaf cotton. This provides the potential to modulate leaf color by altering the expression of this regulatory gene. It will be of great interest to determine how the response to light mediated through this key regulator controls anthocyanin accumulation in cotton leaf.

## Supporting Information

Table S1 **Primers used for gene cloning in this study.**
(DOC)

Table S2 **Primers used for quantitative RT-PCR analysis in this study.**
(DOC)
