# Supplementary material for: The Promoter Structure Differentiation of a MYB Transcription Factor RLC1 Causes Red Leaf Coloration in Empire Red Leaf Cotton under Light
Source: PLoS One. 2013 Oct 29;8(10):e77891. doi: 10.1371/journal.pone.0077891 (PMC3812142; doi:10.1371/journal.pone.0077891)
Supplement: Table S1 — Primers used for gene cloning in this study. (DOC) [file pone.0077891.s001.doc]

**Table S1: Primers used for gene cloning in this study**

| Primer | Forward (5’-3’) | Reverse (5’-3’) |
| --- | --- | --- |
| Degenerate PCR | GAARTATGGGGAAGGTAAGTGG | GTGTTCCARTARTTYTTSACATC |
| 5’-RACE PCR | CA (Kit) | MYB15SP1: ATTCAGCCACCGCAGTCTACAGCTT |
| 3’-RACE PCR | MYB3SP: CCTCCATAACCTCCTAGGTAATAGA | RP (Kit) |
| Genome walking PCR |  | SP1: GCACTTGATGCCATTTCCCTTCAC |
|  | AP1,AP2,AP3 and AP4 (Kit) | SP2: GGTCTTCTTCTTCAGTCCATGCAC |
|  |  | SP3: GCCCTCCATATAGAAGCTGTTATAGC |
| Full *RLC1* cDNA cloning | Ghi2-F2: ATGGAGGGCTCATCTTTA | Ghi2-R2: CTATGGGTTGAACACATTC |
|  | *Sma*Ⅰ-Ghi2-F2: CCCGGGATGGAGGGCTCATCT  TTA | *Sac*Ⅰ-Ghi2-R2: GAGCTCCTATGGGTTGAACACA  TTC |
| *RLC1* alleles cloning | GhDNA-F1: TGGAGGGCTCATCTTTAAGAGTTA | GhDNA-R1: ATACCCATAGTCGTTATTACCGTCG |
|  | GhDNA-F2: CAGATGGTCACTGATTGCTGGT  AGA | GhDNA-R2: CTATGGGTTGAACACATTCCACA  GT |
| Promoter cloning | ProC-F: AAGCTTAGGAGGACTGATTGATATGG CTACA | ProC-R: CCCGGGATAAAAGCTGTTATAGTTAG CTTGC |
