# Supplementary material for: The Promoter Structure Differentiation of a MYB Transcription Factor RLC1 Causes Red Leaf Coloration in Empire Red Leaf Cotton under Light
Source: PLoS One. 2013 Oct 29;8(10):e77891. doi: 10.1371/journal.pone.0077891 (PMC3812142; doi:10.1371/journal.pone.0077891)
Supplement: Table S2 — Primers used for quantitative RT-PCR analysis in this study. (DOC) [file pone.0077891.s002.doc]

**Table S2: Primers used for quantitative RT-PCR analysis in this study**

| Primer | Forward (5’-3’) | Reverse (5’-3’) |
| --- | --- | --- |
| *Gossypium hirsutum* | | |
| GhRLC1 | CAGATGGTCACTGATTGCTGGTAGA | TTCATTCATAGGTTGGTCCAACATG |
| GhCHS | GCTCCGAGTGGCTAAGGACTTA | AACCCTTCTCCTGTGGTCTGA |
| GhCHI | TCACTGTCCGTCACCGAACTC | TGGATTCCAGCACTGAGTTTG |
| GhF3H | GCCAGAGGGTTGGATTGAAGT | GCTTGGGCTTCTCATTCTCAG |
| GhF3'H | ATTCGGCATCCCAACATCATG | CCTCCATGTCCAGCTTCTCAG |
| GhDFR | AACCTGTTGGAGCGACCTTGA | ATGTCTCGACAGCTCCTACGA |
| GhANS | AGCTTGAGTGGGAGGACTACT | GATATGCTGAGCAAAGGTACG |
| Gh3GT | ATATCGCGGATGAGCTGAATA | ATTCCTCCAGGGCTTCACATA |
| UBI7 | GAAGGCATTCCACCTGACCAAC | CTTGACCTTCTTCTTCTTGTGCTTG |
| *Antirrhinum majus* | | |
| AmCHS | GCAGCAGCGGTTATAGTTG | CGCCGAAGACTTCCTCAT |
| AmF3H | TGACTGATATGGCACGAGAGT | TGATCCTGGAGCAGCAAAGTA |
| AmDFR | GTGCGATTGACACTTGCC | CTGCCATCAGTATGATCGTTTG |
| AmANS | GCATTTGATTAACCACGGTGT | CAATAACAACACCACCACCAT |
| AmUBI | ATTGGTGCTGAGGTTGAGA | ACAACTGACTCCAGCAAACG |
